# Periodontal pathogens of the interdental microbiota in a 3 months pregnant population with an intact periodontium

**DOI:** 10.3389/fmicb.2023.1275180

**Published:** 2023-11-01

**Authors:** Florence Carrouel, Aida Kanoute, Virginie-Eve Lvovschi, Denis Bourgeois

**Affiliations:** ^1^Laboratory “Health, Systemic, Process” (P2S), UR4129, University Claude Bernard Lyon 1, Lyon, France; ^2^Public Health Service, Department of Dentistry, Cheikh Anta Diop University, Dakar, Senegal; ^3^Laboratory “Research on Healthcare Performance” (RESHAPE), INSERM U1290, University Claude Bernard Lyon 1, Lyon, France; ^4^Hospices Civils of Lyon, Lyon, France

**Keywords:** gingivitis, microbiota, biofilm, interdental, dysbiosis, periodontal health, pregnancy, periodontitis

## Abstract

Steroid hormones and the oral microbiota of pregnant women both appear as cumulative risk factors for gingivitis. This cross-sectional study, using real-time PCR, investigated the composition and diversity of the microbiota in interdental spaces of 3 months pregnant women with intact periodontium according the 2018 EFP/AAP classification. Bacteria identified were belonged to the red (*Porphyromonas gingivalis Treponema denticola*, and *Tanerella forsythia*), orange (*Fusobacterium nucleatum*, *Prevotella intermedia*, *Campylobacter rectus*, and *Parvimonas micra*), and green (*Eikenella corrodens* and *A. actinomycetencomitans*) Socransky complexes. Approximatively 10^9.11^ bacteria were counted per interdental space in pregnant women. Bacteria from the red complex represented 33.80% versus 62.81% for the orange group versus 3.39% for the green group of the total number spread over the 3 groups. Dietary habits and physical activity did not have a significant impact on interdental microbiota, although a decrease in the median amount of 9 periodontopathogens was observed when fruit and vegetable consumption increased. Pregnant women who brushed their teeth at least twice a day had lower counts of total bacteria and 9 periodontal pathogens than those who brushed less. In 3 months pregnant women at high risk of periodontal disease (>30% bleeding sites), the dendogram revealed 2 clusters of the 9 periodontopathogens. This provides further support for the “key pathogen” hypothesis, among which *Porphyromonas gingivalis* plays a key role, indicating that specific bacteria in limited quantities can influence the host immune system and convert the microbiota from symbiotic to dysbiotic to induce inflammatory disorder. As a result, this study reported that 3 months pregnant women with healthy periodontium had high levels of interdental bleeding and a dysbiotic microbiota with periodontal pathogens of the Socransky orange and red complexes. These subjects were therefore potentially at increased risk of developing periodontal disease and, consequently, an adverse pregnancy outcome. So, preventive oral prophylaxis measures, in particular individual interdental prophylaxis, should be implemented as soon as pregnancy is established.

## 1. Introduction

According to the 2018 EFP-AAP classification of periodontal and peri-implant diseases and conditions, clinical gingival conditions can be observed on an intact periodontium, either in a gingival health or as gingivitis (Caton et al., 2018). However, initial clinical changes from healthy to plaque-induced gingivitis may not be detectable (Murakami et al., 2018). In fact, incipient gingivitis can be considered part of a “clinical health” spectrum, with some sites reporting mild inflammation (Murakami et al., 2018). Chronic gingivitis (ICD-10: K05.1) is a non-specific dental plaque-induced inflammatory condition characterized by gingival inflammation on an intact periodontium, or a reduced periodontium in an individual without a history of periodontitis (Chapple et al., 2018). Gingival bleeding is the most easily identifiable clinical sign of gingivitis (Baudet et al., 2020). Current evidence suggests that oral microbial dysbiosis is a primary etiological factor in gingivitis (Alnasser et al., 2023; Morrison et al., 2023), in which bacterial biofilms on the teeth and gingival tissue play a crucial role (Kharitonova et al., 2021). Moreover, environmental and host susceptibility factors may considerably impact the oral microbiote composition and lead to the onset of lifestyle-related disorders (Saadaoui et al., 2021). The 2018 EFP-AAP classification of periodontal diseases applied to gingivitis mentions local risk factors, known as predisposing factors, and systemic risk factors, so-called modifying factors (Chapple et al., 2018).

Adult interdental spaces – in fact the 30 interdental spaces for an individual – with an intact periodontium are among the most representative local risk factors for gingivitis (Carrouel et al., 2016). These ecological niches facilitate the accumulation, maturation and retention of biofilm. They also restrict access to the oral hygiene practices needed to mechanical disorganization of the biofilm. Similarly, reduced salivary flow in the interdental space has the effect of limiting cleaning of dental and gingival surfaces, and promotes increased gingival inflammation. Recent evidence has revealed that the interdental biofilm of young periodontally healthy subjects is composed of pathogens (*Streptococcus* spp., *Streptococcus mutans*, *Lactobacillus* spp., *Enterococcus* spp., and *Candida albicans*) that are able to induce interproximal caries (Bourgeois et al., 2017; Inquimbert et al., 2019). Virulent periodontal pathogens such as the bacteria of orange and red complex (*Campylobacter rectus* (*C. rectus*), *Prevotella intermedia* (*P. intermedia*), *Parvimonas micra* (*P. micra*), *Fusobacterium nucleatum* (*F. nucleatum*), *Porphyromonas gingivalis* (*P. gingivalis*), *Tannerella forsythia* (*T. forsythia*), *Treponema denticola* (*T. denticola*)) were found in interdental spaces indicating that the periodontal disease process could be initiated (Carrouel et al., 2016; Inquimbert et al., 2019; Kharitonova et al., 2021).

Elevations in sex steroid hormones during pregnancy is considered as a systemic risk factor, negatively influence the immune-inflammatory response to a given dental plaque biofilm burden, resulting in exaggerated or “hyper” inflammation (Chapple et al., 2018). Pregnancy includes a multitude of physiological changes: weight gain, hormonal, and metabolic changes, as well as immune changes (Nuriel-Ohayon et al., 2016). An additional important factor which both influences and is affected by these physiological processes is the oral microbiome (Ye and Kapila, 2021). Gingival tissues contain receptors for the sex hormones, estrogen and progesterone, which are susceptible to hormonal imbalances that occur in women during the pregnancy (Sathish et al., 2022). Increased sensitivity to stimuli occurs in the gingiva during pregnancy, gingival alterations associated to the biofilm formation during pregnancy may increase the severity of gingivitis (Terzic et al., 2021).

Recently, gingivitis was suggested as a new risk factor for systemic inflammation. Numerous correlations have been suggested between periodontal conditions and pregnancy complications and outcomes (Gare et al., 2021; Nannan et al., 2022). Oral microbiome composition can contribute to pregnancy complications (Gare et al., 2021; Saadaoui et al., 2021). The infected periodontal tissues act as a reservoir for bacteria that can transfer from periodontal tissues to the fetal placenta unit and trigger a metastatic infection (Bobetsis et al., 2020; Genco and Sanz, 2020).

Symptoms of gingivitis in pregnancy typically emerge during the first trimester (Giglio et al., 2009). Recent evidence has revealed that gingivitis cases and bleeding were particularly high among 3 months pregnant women. Gare et al., indicated that 93% among 3 months pregnant Senegalese women had at least one site with bleeding and 18.6% had 100% of sites with bleeding. 15% had moderate gingivitis, and 73% had severe gingivitis (Gare et al., 2023). Massoni et al., underlined that progesterone levels in the first trimester are positively correlated with *P. gingivalis*, and this relevance suggests that progesterone levels during this period stimulates *P. gingivalis* growth and elevates gingival inflammation (Massoni et al., 2019).

Current knowledge about the subgingival bacterial environment most closely related to periodontal disease in pregnant women is incomplete (Yang et al., 2019). There is limited research on the composition of the oral microbiome throughout pregnancy. There may be alterations in the oral microbiome during pregnancy such as increased relative abundance of pathogenic bacteria (Koerner et al., 2023). The distribution and clustering of oral microbiome for gingivitis among women at 3 months of pregnancy have not been explored and this especially in the interdental spaces. So, to develop better prediction and intervention approaches for adverse pregnancy outcomes, it is critical to understand the oral microbiome changes during pregnancy (Ye and Kapila, 2021).

The recent 2018 EFP/AAP classification, considered as a “precision medicine” approach, provide standardization for determining which patients with intact periodontium would be clinically diagnosed as having gingivitis in terms of prevalence and severity (Chapple et al., 2018). The cartography of bacteria based on the Socransky complexes, which represent a reference framework for the entire scientific community is a key element (Socransky et al., 1998). Six bacteria complexes often encountered in case of periodontal disease were identified and coded as blue, green, yellow, violet, orange, and red. The yellow, green, blue, and purple complexes were mainly associated with periodontal health whereas orange and red complexes were linked to periodontal disease (Socransky et al., 1998; Socransky and Haffajee, 2005). However, as previously described, recent research on bacterial quantification of gingivally healthy adolescents and young adults has shown a significant proportion of orange and red complex bacteria in the interdental spaces (Carrouel et al., 2016; Inquimbert et al., 2019). Thus, in our study, 9 periodontal bacteria (2 from green complex, 4 from the orange complex and the 3 of the red complex) were analyzed qualitatively and quantitatively by real-time PCR.

The objective of this research was to investigate *in vivo* the composition and diversity of the periodontal subgingival microbiota in interdental spaces of 3 months pregnancy women with an intact periodontium according the 2018 classification standard.

## 2. Materials and methods

### 2.1. Study design and setting

This cross-sectional study is a part of the randomized controlled trial OP-PE protocol published by Kanoute et al. (2021) and registered at ClinicalTrials.gov (NCT04989075). The ethical committee of Dakar (Senegal) approved the protocol on 8 June 2021 (000086/MSAS/CNERS/SP).

The multicentre study occurred in 6 National Hospital Center of Dakar (Senegal) and carried out in conformance with the Declaration of Helsinki. Written informed consent was obtained from each participant before enrolment.

This research was performed in accordance with the STROBE guidelines (Supplementary File 1).

### 2.2. Participants

The population was composed of 100 pregnant women at 3 months who had voluntarily presented at one of the 6 hospitals from Dakar for their first visit for a pregnancy diagnosis between March 2022 and August 2022.

Eligibility was limited to (i) women less than 12 weeks pregnant, (ii) aged 18 to 35 years, (iii) from sub-Saharan Africa, (iv) nulliparous at the time of the obstetric visit, (v) accepting the terms and conditions of the study, and (vi) signing the informed consent form.

The obstetric exclusion criteria concerned pregnant women: (i) with congenital uterine and vaginal anomalies, (ii) who had undergone premature termination of pregnancy for medical reasons, (iii) with fetal distress, and (iv) with infectious or systemic diseases.

The oral exclusion criteria concerned pregnant women: (i) having fewer than 20 natural teeth, except third molars, (ii) having none of the 4 premolar-molar pairs, (iii) having a history or treatment of PD, (iv) undergoing dental or orthodontic treatment, (v) having generalized periodontal lesions (>30% of sites) of stage II, III, IV (PD ≥ 4 mm, and/or CAL ≥ 4 mm), (vi) receiving drugs, including antibiotics, affecting the gingiva and/or oral mucosa, (vii) using dental floss and/or interdental brushes and/or mouthwash regularly, or (viii) unable to provide answers to questions or uncooperative.

### 2.3. Outcomes

#### 2.3.1. Primary outcomes measures

The primary outcome was to quantify the total bacterial load as well as the 9 periodontal bacteria of the interdental microbiota in 3 months pregnant women.

#### 2.3.2. Secondary outcomes measures

The secondary outcomes were to analyze the relationships between demographics, lifestyle factors, oral hygiene habits, oral clinical health status and periodontal pathogens load of the interdental microbiota in 3 months pregnant women.

### 2.4. Procedures

The screening of pregnant women was realized during their first prenatal visit. After presentation of the study, pregnant women who met the inclusion criteria and consented to participate were invited to the inclusion visit scheduled at 3 months of pregnancy.

Included pregnant women signed an informed consent form, completed a questionnaire, and underwent obstetrical, oral clinical examinations and interdental sampling. All this information was recorded on an electronic medical record (e-CRF Voozalyon 1.3; Voozanoo, Caluire, France).

#### 2.4.1. Demographic and behavioral features

Sociodemographic characteristics, oral hygiene habits, lifestyle factors, and medication use were obtained using a questionnaire. Pregnant women were considered to have insufficient vegetable and fruit intake if they declared eating less than five portions per day, so at least 400 g (or 5 servings) of vegetables and fruits per day (Nishida et al., 2004; Wang et al., 2014). Pregnant women who performed less than 150 min of moderate-intensity physical activity (600 metabolic equivalents of task (METs)) per week were classified as insufficiently physically active. Inactivity of less than 150 min of moderate physical activity per week (i.e., 30 min a day, 5 days a week) or 75 min of vigorous physical activity (25 min, 3 days a week) is sedentary (Bull et al., 2020).

#### 2.4.2. Prenatal clinical status evaluation

Measurement of weight (kg) and height (cm) using a stadiometer and a calibrated clinical scale were performed during the obstetric clinical examination. Body mass index (BMI) was calculated. Pregnant women were classified as underweight (BMI < 18.5 kg/m^2^), healthy (18.5 kg/m^2^ ≤ BMI < 25 kg/m^2^), overweight (25.0 kg/m^2^ ≤ BMI < 30 kg/m^2^), or obese (BMI ≥ 30.0 kg/m^2^) (CDC, 2022).

#### 2.4.3. Oral clinical examination

Trained and calibrated periodontists performed the clinical examination. The kappa statistic between different examiners was 0.78 (95% CI: 0.14–1.38). Clinical examinations of the whole mouth, including probing depth (PD), clinical attachment level (CAL), gingival index (GI) and plaque index (PI), were carried out by a practitioner in a clinical center using a sterile Williams PDT probe at 20 g pressure (Zila-Pro-Dentec Inc., Batesville, AR, United States) located parallel to the long axis of the tooth (Löe, 1967).

Bleeding on the interdental brushing index (BOIB) corresponds to the bleeding response to horizontal pressure applied in the interdental area by a calibrated interdental brush (IDB) and was performed as described by Bourgeois et al. (2016). A score of 0 corresponds to no bleeding after 30 s and a score of 1 to bleeding after 30 s (Caton and Polson, 1985; Hofer et al., 2011).

Gingivitis on intact periodontium and gingivitis on reduced periodontium in a patient with no history of periodontitis are defined as bleeding sites ≥10% with probing depths ≤3 mm. Localized gingivitis is defined as 10–30% bleeding sites, and generalized gingivitis as >30% bleeding sites (Trombelli et al., 2018).

The diameter of all interproximal spaces of four pairs of teeth (premolars-molars) was assessed using an IAP CURAPROX^©^ colorimetric probe (Curaden, Kriens, Switzerland). Participants were not to brush, drink, eat or practice oral hygiene for 3 h prior to the sampling visit.

#### 2.4.4. Interdental sampling

For all pregnant women, the same four interdental sites (15–16, 25–26, 35–36, and 45–46) were evaluated (100 sites in total). If one of the sites was missing due to the absence of a tooth, the adjacent medial site was sampled as a replacement. The appropriate interdental brushes (Curaden, Kriens, Switzerland) were determined during the clinical assessment of the interdental spaces (Bourgeois et al., 2015). Selected teeth were isolated using sterile cotton rolls. Interdental biofilm was collected using a sterile, calibrated interdental brush (IDB) introduced and then removed from the interdental space (Carrouel et al., 2016). The 4 IDBs from one pregnant woman were pooled into 1 sterile 1.5 mL microcentrifuge tube and stored at 4°C for further processing.

#### 2.4.5. Microbiological analysis

##### 2.4.5.1. DNA extraction

Total DNA was extracted from IDBs using the QIAcube HT Plasticware and Cador Pathogen 96 QIAcube HT Kit (Qiagen, Hilden, Germany), according to manufacturer’s guidelines. Using UV at 260 and 280 nm, the quality and quantity were measured.

##### 2.4.5.2. Quantification of periodontal pathogens from the interdental microbiota

To quantify the total bacterial load (TB) and that of 9 pathogen species (*E. corrodens, A. actinomycetemcomitans, C. rectus, P. intermedia, P. micra, F. nucleatum, P. gingivalis, T. forsythia, T. denticola*) present in the biofilm interdental samples, the method described previously was used (Carrouel et al., 2016).

#### 2.4.6. Statistical analysis

The statistical analysis consisted of three main steps: producing descriptive summaries of the data, modeling the data using a mixed (linear) model and assessing the correlations between bacterial abundances. SPSS Windows 20.0 (IBM, Chicago, IL, United States) was used for the descriptive statistics median values and interquartile range (IQR) and mean values with SD for the quantitative variables and percentages for categorical variables. Microbiological variables will be presented as total periodontal bacteria counts, frequency of detection of target pathogens, counts of each pathogen studied, and proportions of each pathogen in the total microbiota. Total periodontal counts will be log-transformed to match a normal distribution.

Descriptive bivariate analyses between participant characteristics and the outcome, gingivitis, were evaluated using *t*-tests and logistic regression for continuous and binary/categorical variables, respectively. Kruskal–Wallis tests were performed to compare the mean counts for the different bacterial species relative to each clinical characteristic. The detailed statistical methods are indicated in the table footnotes. Shapiro–Wilk test was used to check the normality of the distribution. Results will be considered statistically significant at *p* < 0.05.

Principal component analysis (PCA) was performed to identify clusters of variables highly associated with each other and to visually assess whether groups with and without these outcomes of interest could be distinguished using the variables identified in both univariate and multivariate analyses. Briefly, the dataset was dimensionally reduced into linear variables termed principal components (PCs) with those possessing eigenvalues >1 included in further analyses. Loadings were computed against PCs to determine correlations, which were visualized in PC biplots. Using matplotlib.pyplot (V.3.7.1) in Jupyter Notebooks. A Min-Max normalization was done for the non-(0-1) variables. A correlation circle was used to figure the results. The main idea of these analyses is thus to assess whether pregnant women with and without a given outcome of interest may be regrouped within two different groups or, on the contrary, cannot be distinguished.

## 3. Results

### 3.1. Characteristics of pregnant women

**Figure 1:**
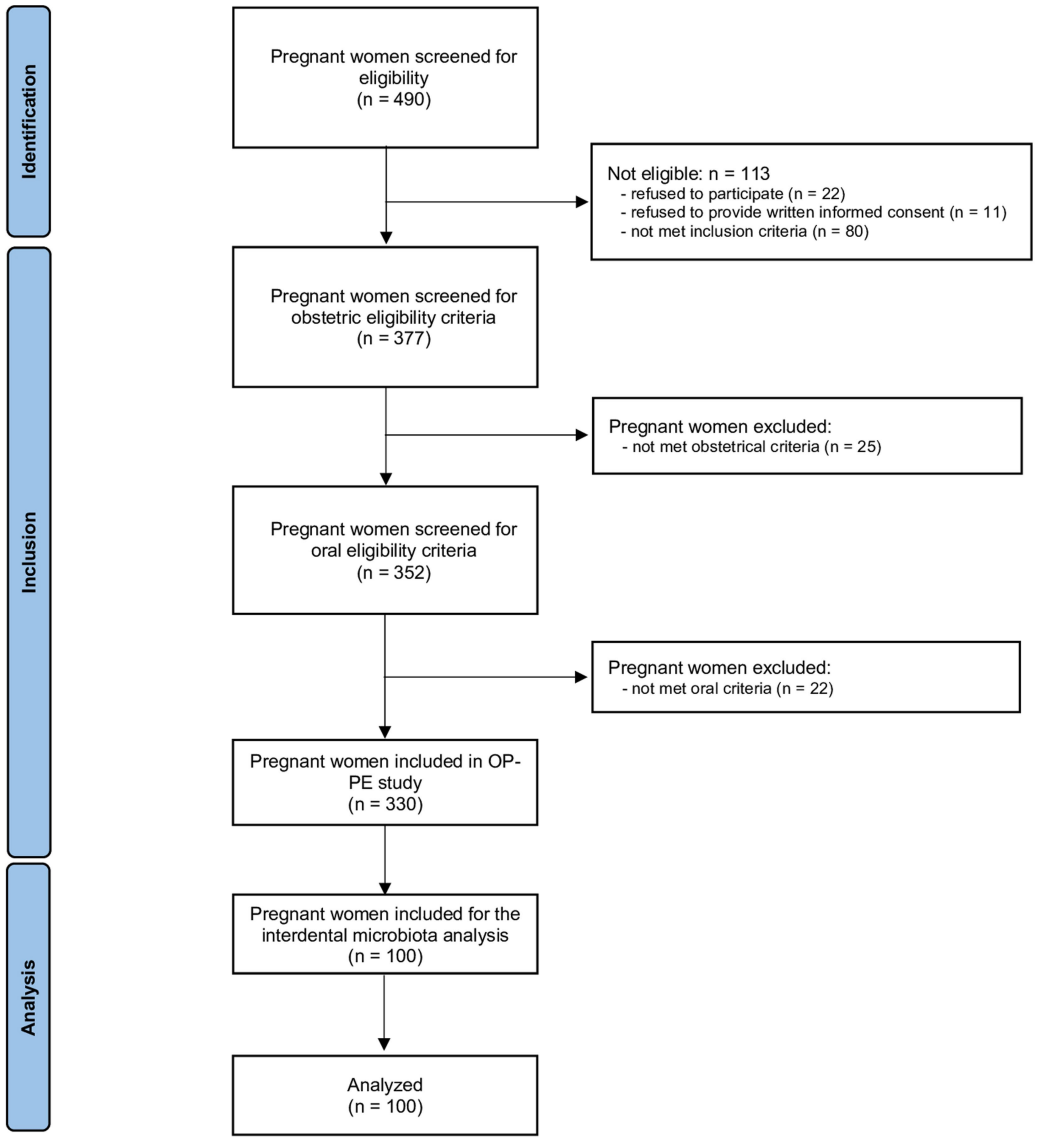
Flow chart of the study.

The flow chart is presented in Figure 1. Of the 490 pregnant women who was accessed for eligibility, 113 were ineligible, 25 did not meet obstetrical inclusion criteria and 22 did not meet oral inclusion criteria. Among the 330 women included in OP-PE study, 100 were randomly selected to analyse the interdental microbiota and are included in this study.

#### 3.1.1. Demographic characteristics

Table 1 presents characteristics of the pregnant women. Among the 100 pregnant women included, the median age was 23.0 [20.0–26.0] years. Of these, 94.0% (*n* = 94) were urban dwellers, 20.0% (*n* = 20) had never been to school, and 61.0% (*n* = 61) had no professional activity. No women were current smokers and 1% (*n* = 1) reported harmful alcohol use. The pregnant women had a median BMI of 22.55 Kg/m^2^ (IQR 20.3–26.3). Among them, 12.0% (*n* = 12) were classified as underweight, 58.0% (*n* = 58) normal, and 30.0% (*n* = 30) obese. In addition, 82.0% (*n* = 82) declared consumed less than 5 fruits and vegetables per day and 70.0% (*n* = 70) reported a sedentary lifestyle during the first 3 months of pregnancy. In terms of oral hygiene, 30% (*n* = 30) of participants reported cleaning their teeth at least twice a day with a toothbrush, 87% (*n* = 87) reported using a toothbrush alone, 1% (*n* = 1) a dental stick and 12% (*n* = 12) both.

**Table 1:** Demographic characteristics and lifestyle habits of pregnant women.

Characteristics	Overall (*N* = 100)
Age (years)	
mean ± SD	23.7 ± 4.6
median [IQR]	23.0 [20.0–26.0]
Pregnancy weeks	
mean ± SD	12.2 ± 0.7
median [IQR]	12.0 [12.0–13.0]
Professional activity, *n*/*N* (%)	
No	61/100 (61.0)
Yes	39/100 (39.0)
Residence, *n*/*N* (%)	
Urban	94/100 (94.0%)
Rural	6/100 (6.0%)
BMI (Kg/m^2^)	
mean ± SD	23.63 ± 4.89
median [IQR]	22.55 [20.3–26.3]
Education, *n*/*N* (%)	
No	20/100 (20.0%)
1–6 years	21/1000 (21.0%)
7–12 years	24/100 (24.0%)
≥13 years	35/100 (35.0%)
Fruits and vegetables consumption, *n*/*N* (%)	
<5 portions by day	82/100 (82.0)
≥5 portions by day	18/100 (18.0)
Physical activity, *n*/*N* (%)	
No <1,500 metabolic equivalent of task/week	85/100 (85.0)
Yes ≥1,500 metabolic equivalent of task/week	15/100 (15.0)
Sedentary behavior, *n*/*N* (%)	
Active	30/100 (30.0)
Sedentary	70/100 (70.0)
Toothbrushing frequency, *n*/*N* (%)	
<2/day	70/100 (70.0)
≥2/day	30/100 (30.0)

#### 3.1.2. Clinical characteristics

The clinical parameters of the pregnant women are described in Table 2. In 32.0% of cases, pregnant women had sound teeth (no cavities, no fillings, no missing teeth due to cavities). The DMFT index was very low (<3) for 86.0% (*n* = 86) of pregnant women. More than 1 decayed tooth was observed for 37.0% (*n* = 37) of cases and one or more missing teeth was reported for 30% (*n* = 30) of cases. Conservative care was observed for 9.0% (*n* = 9) of pregnant women.

**Table 2:** Clinical parameters of pregnant women.

Characteristics	Overall (*N* = 100)
Caries free, *n*/*N* (%)	
Yes	32/100 (32.0)
No	68/100 (68.0)
Decayed teeth	
mean ± SD	1.8 ± 2.5
median [IQR]	1.0 [0.0–2.0]
Number of teeth	
mean ± SD	27.0 ± 2.8
median [IQR]	28.0 [27.0–28.0]
Filled teeth	
mean ± SD	0.2 ± 0.6
median [IQR]	0.0 [0.0–0.0]
Plaque index	
mean ± SD	0.57 ± 0.48
median [IQR]	0.45 [0.14–1.0]
Clinical attachment level (mm)	
mean ± SD	1.98 ± 1.08
median [IQR]	2.2 [1.61–2.64]
Bleeding on interdental brushing (%)	
mean ± SD	0.53 ± 0.32
median [IQR]	0.54 [0.29–0.82]

Interdental bleeding was described at least once for 94.0% of the pregnant women, and the median was 54.0% (IQR 29.0–82.0). While 7% of the women had 100% of the sites bleeding, 8% had no bleeding on interdental brushing. Gingivitis (gingival bleeding score ≥10%) was present in 87.0% of pregnant women, generalized gingivitis in 14.0%, and localized gingivitis in 73.0%.

### 3.2. Quantification of the total number of bacteria and 9 periodontopathogens

#### 3.2.1. Quantification of Socransky complexes

**Figure 2:**
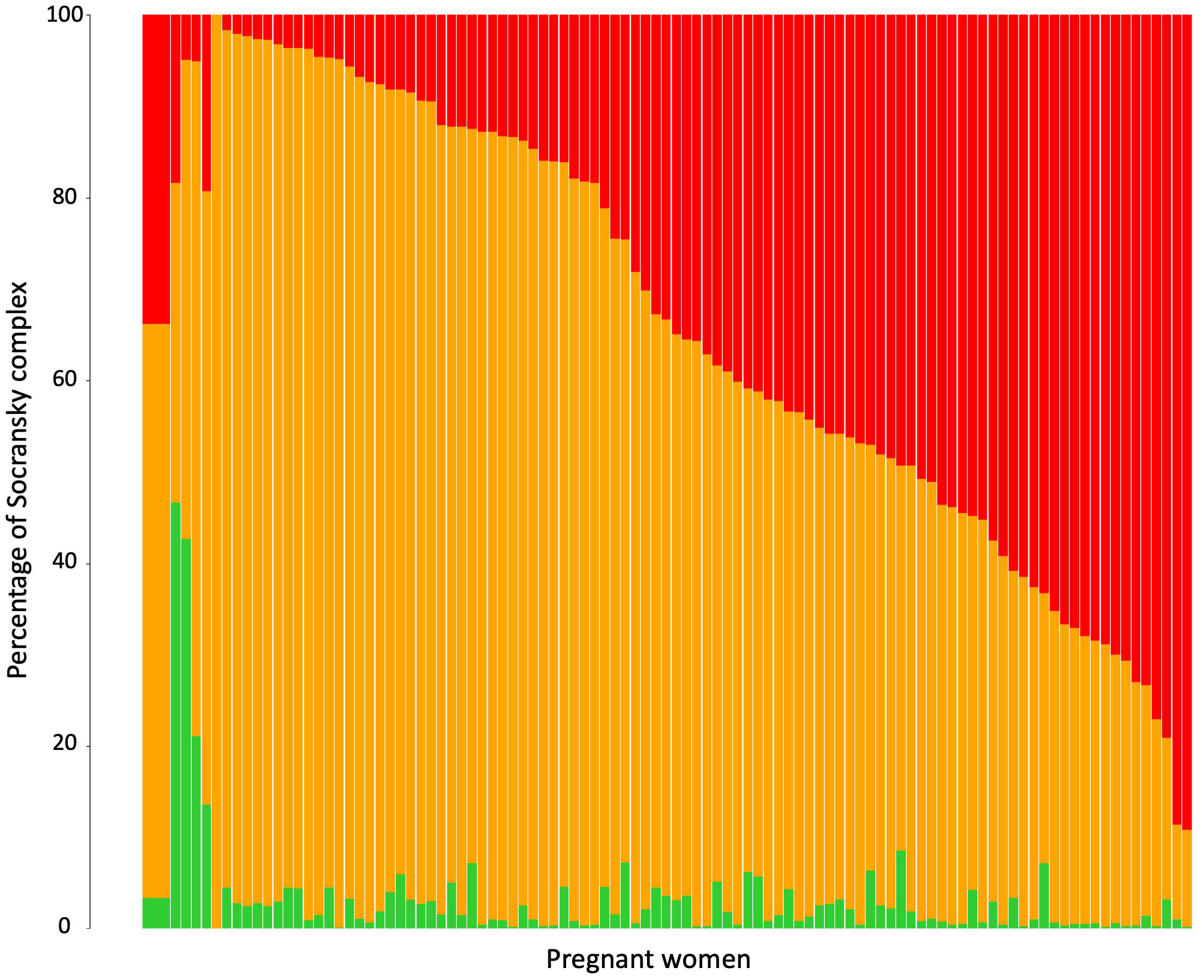
Relative abundance of Socransky complexes among the pregnant women. Percentage of Socransky complex = Quantity of periodontopathogens for one Socransky complex/Quantity of the 9 periodontopathogens. The first bar displays the average proportion of each complex in all pregnant women included. The other bars display the average proportion of each complex in one pregnant woman. The colors refer to the colors of the Socransky complexes: Green = Ec + Aa, Orange = Cg + Cr + Pi + Pm + Fn, and Red = Pg + Tf + Td. Avg, average.

The 9 periodontopathogens analyzed in this study were grouped according to the green, orange and red complexes defined by Socransky et al. (1998). The percentages of each of these complexes per pregnant women were analyzed (Figure 2). The main complex was the orange complex, which represents an average of 62.81%, followed by the red complex (33.80%) and the green complex (3.39%). The percentage of each complex depend on the women. One woman did not have bacteria from the red complex whereas for 26 women the red complex represented more than 50% of periodontopathogens tested.

#### 3.2.2. Quantification of periodontopathogens in relation to demographic, behavioral, and clinical parameters

Table 3 shows the mean total bacterial load and that of the 9 periodontopathogens in the interdental microbiota of pregnant women. On average, the microbiota of an interdental site consisted of 10^9.11^ bacteria. Bacteria from the orange and red complexes were present in greater quantities (From 10^6.38^ for *C. rectus* to 10^7.35^ for *P. micra*) than those from the green complex. *F. nucleatum* and *C. rectus* were present in all the pregnant women whereas *P. intermedia* was present in 51% of women. No significant effect of age, fruits and vegetables consumption, metabolic equivalent of task/week (MET) or body mass index (BMI) factors was observed.

**Table 3:** Average abundances for total number of bacteria and 9 periodontopathogens in various subgroups of the cohort of pregnant women.

Variable	*n*	TB	Ec	Aa	Cr	Pi	Pm	Fn	Pg	Tf	Td
Total^a^	100	9.11 ± 8.97	6.23 ± 6.47	4.95 ± 5.44	6.38 ± 6.83	6.67 ± 7.1	7.35 ± 7.34	7.12 ± 7.29	7.28 ± 7.68	6.90 ± 6.92	7.04 ± 7.31
Positives women^b^	100	100	98	74	100	51	99	100	76	95	93
Age											
18–23^a^	31	9.13 ± 8.9	6.28 ± 6.49	4.99 ± 5.42	6.28 ± 6.45	6.62 ± 6.91	7.38 ± 7.31	7.16 ± 7.39	7.26 ± 7.67	6.91 ± 6.94	7.06 ± 7.21
≥23^a^	31	9.09 ± 9.02	6.17 ± 6.45	4.88 ± 5.46	6.47 ± 6.98	6.72 ± 7.22	7.31 ± 7.38	7.07 ± 7.08	7.29 ± 7.69	6.89 ± 6.89	7.02 ± 7.39
Body mass index											
<18.5^a^	12	9.09 ± 8.98	5.8 ± 5.72	5.0 ± 5.48	6.24 ± 6.28	6.59 ± 6.97	7.39 ± 7.41	6.97 ± 6.85	7.18 ± 7.44	6.9 ± 6.91	7.0 ± 7.23
18.5–25^a^	58	9.14 ± 9.0	6.32 ± 6.55	4.9 ± 5.42	6.45 ± 6.94	6.77 ± 7.18	7.39 ± 7.37	7.16 ± 7.36	7.3 ± 7.7	6.92 ± 6.93	7.09 ± 7.37
≥25^a^	30	9.06 ± 8.88	6.13 ± 6.32	5.01 ± 5.46	6.27 ± 6.5	6.41 ± 6.81	7.25 ± 7.24	7.09 ± 7.19	7.26 ± 7.7	6.87 ± 6.91	6.94 ± 7.13
Fruits and vegetables consumption											
<5^a^	82	9.1 ± 8.99	6.27 ± 6.51	5.02 ± 5.48	6.42 ± 6.87	6.61 ± 7.07	7.37 ± 7.35	7.13 ± 7.32	7.35 ± 7.72	6.92 ± 6.94	7.06 ± 7.33
≥5^a^	18	9.14 ± 8.78	6.02 ± 5.99	4.21 ± 4.58	6.11 ± 6.2	6.87 ± 7.19	7.26 ± 7.31	7.05 ± 7.08	6.63 ± 6.91	6.78 ± 6.78	6.93 ± 7.19
Sedentary											
<150^a^	52	9.16 ± 8.89	6.19 ± 6.31	4.92 ± 5.45	6.5 ± 6.96	6.8 ± 7.2	7.34 ± 7.26	7.11 ± 7.14	7.37 ± 7.74	6.92 ± 6.92	7.1 ± 7.35
≥150^a^	48	9.05 ± 9.02	6.27 ± 6.57	4.98 ± 5.43	6.19 ± 6.39	6.45 ± 6.87	7.36 ± 7.41	7.13 ± 7.39	7.15 ± 7.59	6.88 ± 6.92	6.97 ± 7.25
MET											
<1500^a^	85	9.13 ± 8.98	6.28 ± 6.5	4.98 ± 5.47	6.42 ± 6.86	6.72 ± 7.13	7.32 ± 7.24	7.09 ± 7.12	7.3 ± 7.7	6.9 ± 6.93	7.06 ± 7.33
≥1500^a^	15	9.0 ± 8.81	5.83 ± 5.76	4.64 ± 5.07	6.07 ± 6.22	5.99 ± 6.18	7.5 ± 7.59	7.25 ± 7.61	7.11 ± 7.45	6.91 ± 6.83	6.87 ± 7.0
Toothbrushing frequency											
<2 times per day^a^	70	9.15 ± 9.0	6.22 ± 6.42	5.02 ± 5.49	6.48 ± 6.9	6.74 ± 7.16	7.41 ± 7.39	7.19 ± 7.35	7.36 ± 7.72	6.95 ± 6.9	7.11 ± 7.36
≥2 times per day^a^	29	8.99 ± 8.81	6.25 ± 6.58	4.72 ± 5.19	5.96 ± 6.15	6.24 ± 6.52	7.19 ± 7.1	6.89 ± 6.91	6.98 ± 7.55	6.77 ± 6.94	6.83 ± 7.07
Bleeding											
<30%^a^	27	9.15 ± 9.11	6.35 ± 6.56	4.87 ± 5.43	6.46 ± 6.7	6.75 ± 7.27	7.25 ± 7.2	7.23 ± 7.22	7.28 ± 7.75	6.82 ± 6.89	6.91 ± 7.33
≥30%^a^	73	9.09 ± 8.87	6.18 ± 6.43	4.97 ± 5.44	6.35 ± 6.87	6.63 ± 6.98	7.38 ± 7.38	7.07 ± 7.31	7.28 ± 7.65	6.93 ± 6.93	7.08 ± 7.3

The first two columns correspond to the size of the subgroup and the mean abundance of the total bacterial. The other columns correspond to the mean abundance of each periodontopathogen. The colors refer to the colors of the Socransky complexes. The line “Positives women” analyzes the proportion of women expressing one periodontopathogen. MET, metabolic equivalent of task/week; *n*, number of women; TB, total bacterial load.

a Mean log-count ± standard deviation.

b Percentage.

**Figure 3:**
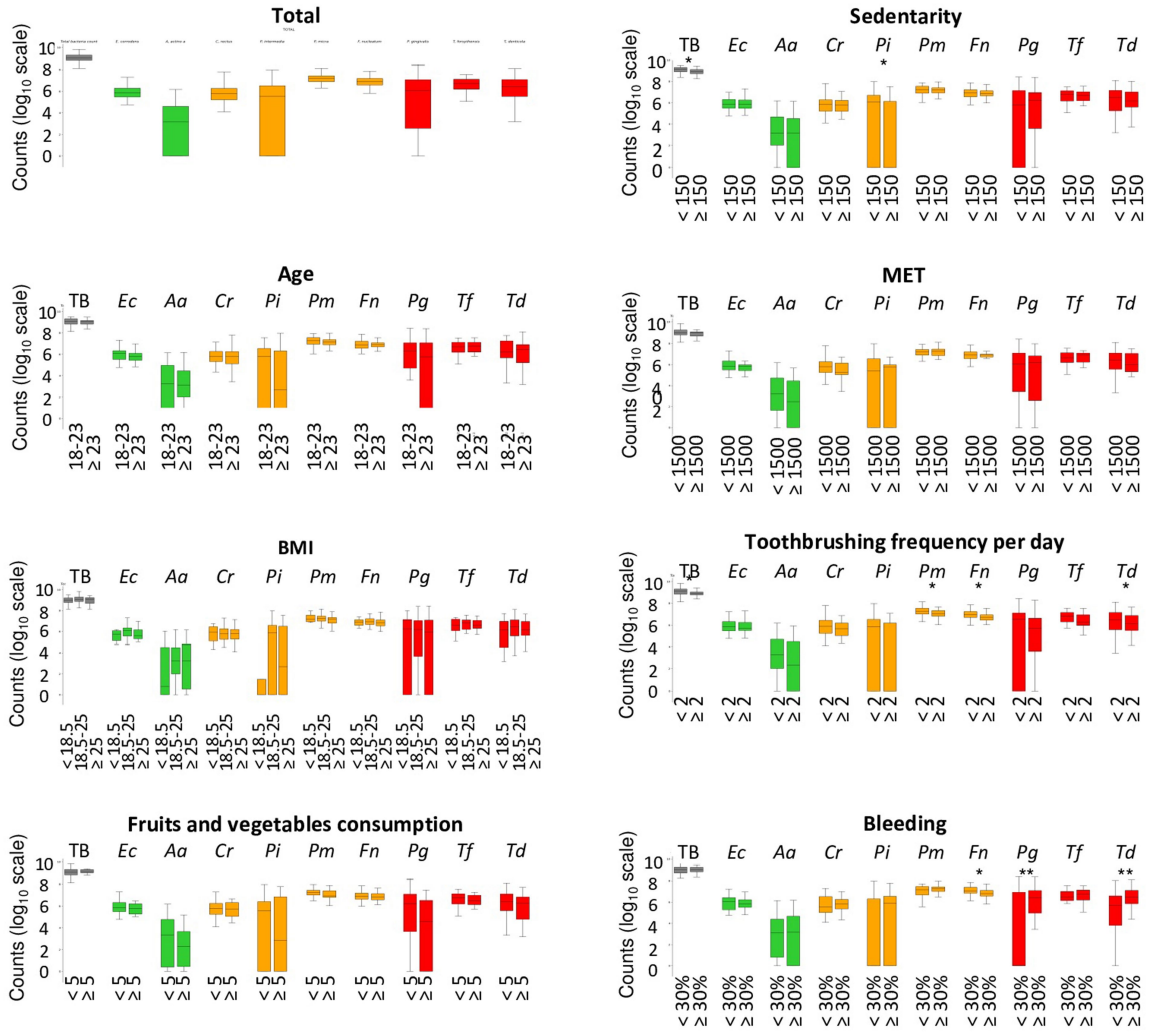
Abondance of periodontopathogens among pregnant woman in relation to demographic, clinical, and behavioral parameters. Systematic significance tests were performed between the periodontopathogen abundances and the tested experimental factors (age, body mass index, fruits, and vegetables consumption, MET, Sedentary, toothbrushing frequency, and bleeding). The stars indicate that the test is significant (*p < 0.05) and highly significant (**p < 0.01). MET, metabolic equivalent of task/week; TB, total bacteria; TG, total of bacteria from the green complex (Ec + Aa); TO, total of bacteria from the orange complex (Cr + Pi + Pm + Fn); TR, total of bacteria from the red complex (Pg + Tf + Td).

Figure 3 and Supplementary Figure S1 presents the repartition of the abundance of total bacteria and 9 periodontopathogens. Concerning the age, the BMI, fruits, and vegetables consumption, sedentarity and MET did not impact significantly on the quantity of bacteria. However, a decrease of the median quantity of the 9 periodontopathogens with the increase of consumption of fruits and vegetable consumption was observed. The average of TB and *P. intermedia* significantly higher in sedentary pregnant women than in active women. Pregnant women who report brushing their teeth at least twice a day have a significantly lower number of total bacteria, *P. micra*, *F. nucleatum*, and *T. denticola*. The number of other periodontopathogens was also reduced but not significantly. Concerning the bleeding, pregnant women with more than 30% of bleeding, have a significant lower quantity of the orange complex bacteria and *F. nucleatum*, but a very significant higher quantity of red complex bacteria, *P. gingivalis* and *T. denticola*.

#### 3.2.3. Dimension reduction and clustering by PCA in the orange and red periodontopathogens subgroups

**Figure 4:**
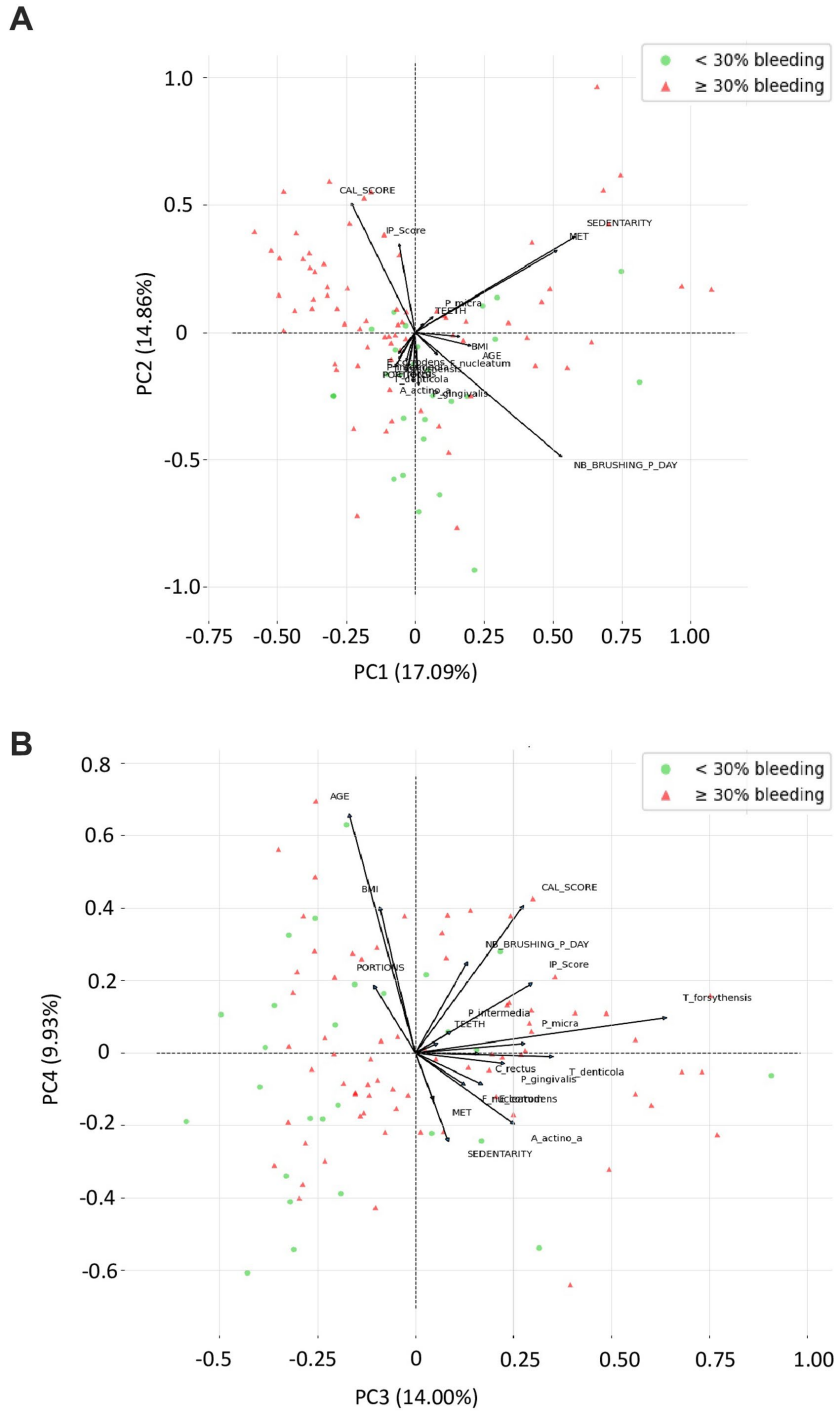
Variable factor map from the principal component analysis (PCA) applied to demographic, behavioral, clinical parameters, and periodontopathogens from the orange and red complexes. These two correlations circles are a visualization displaying how much the original active variables are correlated with the first two principal components. Moreover, bleeding <30% (green dots) and bleeding ≥30% (red dots) are the active observations represented according to the first 2 components computed using principal component analysis of Scikit-Learn. In this PCA, on each correlation circle, active variable is represented by vectors in a 2-dimensional space in such a manner that the closer 2 vectors are, the more women share similar characteristics. If 2 arrows are close, the angle between them is close to 0 and Cos (0°) = 1, so their correlation coefficient is close to 1. If 2 arrows are perpendicular, then the angle between them is 90° and Cos (90°) = 0, so their correlation coefficient is close to 0. BMI, body mass index; CAL_score, clinical attachment loss score; IP_score, plaque index score; MET, metabolic equivalent of task/week; Nb_brushing_p_day, number of toothbrushing per day; Portions, vegetable and fruit intake per day; SEDENTARY, Sedentary behavior; Teeth, number of teeth.

Figure 4 presents the correlation plot from the principal component analysis (PCA) applied to demographic, behavioral, clinical parameters, and periodontopathogens from the orange and red complexes. A Bartlett sphericity test was performed and assessed the variables were dependent (value of *p* < 0.0001). The Kaiser–Meyer–Olkin overall measure of sampling adequacy was 0.51, which indicate a moderate level of sampling adequacy for PCA. The PCA revealed 4 principal components accounted for 55%, 88% of the overall variation of the data produced from the 19 active variables. The first 4 PCs, representing 17,09%, 14,86%, 14,00%, and 9,93% variance respectively, were included in PCA correlations with bleeding percentage; determined using Cattell’s scree plot. Two biplots (correlation circles) were chosen (Figures 4A,B), one according to PC1 and PC2 accounting for 31,95% of the total variations and one according to PC3 and PC4 accounting for 23,93% of the total variations considering a relative low contribution of each component. One third of the vectors in both biplots are of modest size, so the results regarding these variables must be taken with caution.

According to Figure 4A, *C. rectus*, *E. corrodens*, and *A. actinomycetemcomitans* are nearly 100% positively correlated with PC1, while, sedentarity, IP score are highly negatively correlate to PC2, and while *P. micra*, *T. forsythensis*, *F. nucleatum*, *T. denticola* tend to be correlated with a combination of PC1 and PC2. PC1 seem to be highly linked to bacterial identification, as PC2 seem to be more related to pregnant women behavior. BMI, age, and no teeth brushing had low contribution. Bleeding lower than 30% seem to be highly dispersed. *E. corrodens* and *C. rectus* seem to be almost collinear, as MET and sedentarity with their respective PC.

According to Figure 4B, CAL score seems to be nearly 100% negatively correlated with PC3, as portions with PC4. Age, BMI and the frequency of tooth brushing per day, tend to be negatively correlated with a combination of PC3 and PC4, as sedentarity and MET tend to be positively correlated with a combination of PC3 and PC4. PC3 seem to be highly linked to pregnant women behavior, as PC4 seem to be more related to pregnant women socio-demographic characteristics. Most bacteria had low contribution. In this projection, bleeding <30% seem to be more frequent when variables are highly correlates with PC3, but we cannot formulate a clear hypothesis about pregnant behavior as involved in bleeding occurrence considering discrepancy with first biplot.

#### 3.2.4. Correlation of periodontopathogens in relation to the bleeding

**Figure 5:**
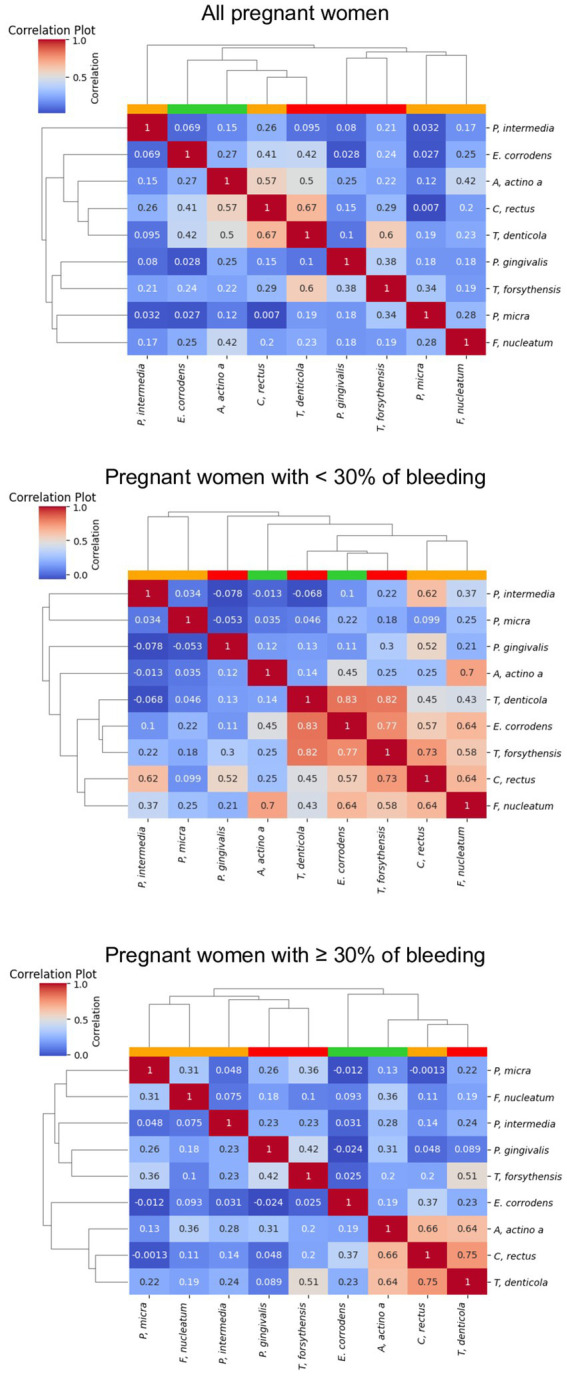
Correlation plot of the abundance of the periodontopathogens. The red, white, blue squares indicate positive, zero, negative correlations, respectively. The colored leaves on the top dendrogram represent the color of Socransky complexes.

Figure 5 shows the correlations between the 9 periodontopathogens and the interdental site of the 100 pregnant women according to the percentage of bleeding. The dendogram analyzing all the pregnant women underlines the presence of 2 clusters but with a low correlation between bacteria. The first is composed of bacteria from the orange and red complexes (*F. nucleatum*, *P. micra*, *T. forsythia* and *P. gingivalis*). The second is composed of the bacteria from the green, orange and red complexes (*C. rectus*, *E. corrodens*, *T. denticola*, *A. actinomycetemcomitans*, and *P. intermedia*). The 3 bacteria of the red complex belong to 2 different clusters but are in close contact. For pregnant women presenting less than 30% of bleeding, a cluster composed of 5 highly correlated periodontopathogens (*F. nucleatum*, *C. rectus*, *T. forsythia*, *E. corrodens*, and *T. denticola*) appears. For pregnant women presenting more than 30% of bleeding, periodontopathogens are organized in 2 clusters. The first one is composed of bacteria from the orange complex (*F. nucleatum*, *P. micra*, and *P. intermedia*) and the red complex (*P; gingivalis* and *T. forsythia*) but the correlation is low between bacteria. The second one is composed of bacteria of the green, orange and red complexes and underlines a high correlation between *T. denticola*, *C. rectus*, *A. actinomycetemcomitans* and a low correlation with *T. forsythia*.

## 4. Discussion

To our knowledge, this study is the first to analyze qualitatively and quantitatively the total number of bacteria and 9 periodontopathogens present in the interdental microbiota of 3 months pregnant women with intact periodontium according the 2018 classification standard (Chapple et al., 2018). A characterization of the oral microbiota of pregnant women with gingivitis could greatly help to establish effective ways to prevent and treat these diseases and decrease risk factors conducing to adverse pregnancy outcomes (Jang et al., 2021). More particularly, it is important to analyze the interdental microbiota of pregnant women because previous studies have demonstrated that the interdental space is a reservoir of periodontopathogens, even in healthy young people without clinical signs of periodontal disease (Carrouel et al., 2016).

Gingivitis – plaque induced – in our study was present in 87.0% of 3 months pregnant women. If classically, epidemiologic data have shown plaque-induced gingivitis to be prevalent at all ages in dentate populations (Mostafa and El-Refai, 2018), we must be aware that additional researches were necessary to obtain standardized information due to the heterogeneity of these studies including different target group and in a context where the definition of gingivitis prior to the 2018 classification was unclear (Gare et al., 2023). Characterizing the oral microbiome of healthy gingival pregnant women is an effective initial stage to understand the role of the microbiome in the progression of pregnancy-induced gingivitis (Balan et al., 2021). As mentioned by Murakami et al. (2018), it must be considerate that the intensification of the inflammatory gingival response, whether due to changes in sex steroid hormone secretions or pathogenic biofilms, represent an individual’s protective responses to systemic and local environments by destroying, attenuating and isolating invasive microorganisms (Murakami et al., 2018).

Our study indicated that one interdental space of pregnant women contains approximatively 10^9.11^ bacteria. For information, in periodontally healthy young adults aged 20–35, an interdental site contained 10^10^ bacteria (Carrouel et al., 2016) and in periodontally healthy adults aged 35–56, the supragingival biofilm of the interdental space contained 10^7.56^ bacteria (Field et al., 2012).

Periodontopathogens were identified in interdental microbiota of pregnant women. The 9 periodontopathogens are organized into clusters with a similar organization that the complexes of Socransky et al. (1998). The periodontopathogens were present in pregnant women (green complex: *E. corrodens* in 98% of pregnant women and *A. actinomycetencomitans* in 74% of pregnant women); orange complex: *P. intermedia* in 51% of pregnant women; *P. micra* in 99% of pregnant women; *C. rectus* and *F. nucleatum* in 100% of pregnant women; red complex: *P. gingivalis* in 76% of pregnant women; *T. denticola* in 93% of pregnant women; *T. forsythia* in 95% of pregnant women. Pregnant women (23.66 ± 4.62 years old) compared to periodontally healthy young adults (mean age of 26.8 ± 4.6 years old) (Carrouel et al., 2016) have a higher quantity of *P. gingivalis* (10^7.28^ versus 10^3^ in one ID space), *T. denticola* (10^6.9^ versus 10^6^ in one ID space), and *T. forsythia* (10^7.28^ versus 10^3^ in one ID space). The increasing of periodontal bacteria could be explained by the pregnancy. Indeed, during pregnancy, hormonal and metabolic fluctuations have a systemic action on cellular and immunological levels in order to respond and adapt to the demands of the developing fetus. Maternal hormonal changes (estrogen and progesterone) during pregnancy modify the maturation and activation of immune cells against infectious organisms (Gibbs, 2001). A reduced activation of maternal peripheral lymphocytes was observed when subjected to antigens and, compared to non-pregnant or male subjects, altered maternal estrogen and progesterone levels increased and suppressed prostaglandin E2 and interleukin-1β, respectively (Ortiz-Sánchez et al., 2021). Thus, the link between high maternal hormone levels and increased numbers of periodontal pathogenic bacteria, could be influenced directly and indirectly, facilitating the entry and multiplication of pathogenic bacteria (AlSharief and Alabdurubalnabi, 2023). Regarding the direct pathway, Gibbons et al. demonstrated that pathogens, in particular *P. intermedia* and *P. gingivalis*, produce vitamin K from these hormones, which is vital for bacterial growth (Gibbons and Macdonald, 1960). With regard to the indirect pathway, increased hormone levels altered gingival clinical parameters with increased probing depth and greater quantity and rate of gingival crevicular fluid, affecting the quality and quantity of marginal gingival keratinization and diminishing the immune response (Lindhe and Brånemark, 1967). In a previous study, Carrillo-de-Albornoz et al. (2010) demonstrated that higher progesterone levels in salivary samples were significantly associated with *P. gingivalis* bacterial counts (Carrillo-de-Albornoz et al., 2010). In addition, the presence of periodontopathogens of the orange and red complex that are considered as major etiologic agents of periodontal disease (Mohanty et al., 2019) indicates that pregnant women are at risk of adverse outcomes (Gare et al., 2023). This may be explained by the fact that hormonal changes in periodontal tissues lead to changes in connective tissue cell turnover rates, a decrease in vascular response and an increase in gingival vascular permeability. This creates a pathway for periodontal pathogens to infiltrate and invade the circulation, establishing infections in feto-placental units (Wu et al., 2015).

In addition, in case of gingivitis, the 2018 EFP-AAP classification of periodontal diseases mentions local risk factors, known as predisposing factors, and systemic risk factors, so-called modifying factors (Chapple et al., 2018). These environmental and host susceptibility factors can impact on the symbiosis of the oral microbiome and lead to the onset of lifestyle-related disorders (Saadaoui et al., 2021; Sedghi et al., 2021). Regarding the total count of bacteria and the 9 periodontopathogens, our study indicated that demographic parameters, eating habits and physical activity did not impact significantly on the interdental microbiota. However, a decrease of the median quantity of 9 periodontopathogens with the increase of consumption of fruits and vegetable consumption was observed. This data could be correlated to the fact that people adopting a ‘healthy diet’ have a reduced risk of non-communicable diseases (Martinon et al., 2021). Laiola et al. (2020) demonstrated that a Mediterranean diet in a group overweight/obese subjects led to the significant decreasing of periodontal pathogens such as *P. gingivalis*, *P. intermedia*, and *T. denticola* in the saliva (Laiola et al., 2020). Furthermore, the extract of phenolic-rich cranberry (0.1–1.0 mg/mL) demonstrated a significant antibiofilm activity acting on bacterial adhesion in the early stages of biofilm development, as found in *F. nucleatum*, *P. gingivalis*, and *A. actinomycetemcomitans* (Sánchez et al., 2020).

Contrary to previous factors analyzed in this study, the oral hygiene behavior and more particularly the frequency of toothbrushing impact on the quantity of bacteria Socransky’s complexes. The quantity of the total number of bacteria and of the 9 periodontopathogens decreased in pregnant women brushing their teeth at least 2 times per day compared to pregnant women who brushed less. More particularly, the decrease was statistically significant for the total number, *P. micra*, *F. nucleatum*, and *T. denticola*. This is in accordance with the review of Rajwani et al. (2020) who concluded that toothbrushing promote plaque removal and reduce gingivitis (Rajwani et al., 2020). However, as the toothbrush cannot enter in the interdental space, toothbrushing acts on supragingival plaque but not on the interdental microbiota. Thus, it is necessary to implement oral health education program and more particularly, to educate pregnant women to the interdental cleaning using calibrated interdental brushes (Kiger et al., 1991; Bourgeois et al., 2015). Indeed, several studies have demonstrated that daily use of interdental brushes reduces the dysbiosis of the interdental microbiota, the interdental bleeding and therefore the risk of PD and other non-communicable diseases (Bourgeois et al., 2016, 2019). Particularly in pregnant women, it is important to reduce the risk of adverse pregnancy outcomes due to periodontal bacterial and there, public health policies based on individual oral prophylaxis should be implemented.

In terms of clinical signs, in pregnant women, the percentage of bleeding sites was associated with the plaque index that is to say the quantity of bacteria. This is in concordance with the definition of gingivitis caused by plaque (Chapple et al., 2018). Gingival tissue is characterized by redness, swelling, sensitivity, a shiny surface and bleeding on gentle probing. Generally painless and without spontaneous bleeding, gingivitis often goes undiagnosed (Trombelli et al., 2018). However, in our study, 92% of pregnant women suffered of bleeding. Focusing on pregnant women with more than 30% bleeding revealed, via dendogram observation, a different organization of bacterial clusters to that observed for women with less than 30% bleeding. The 2 clusters organization reveals a high risk to develop periodontal disease. In fact, the first cluster was composed of *F. nucleatum*, *P. micra*, *P. intermedia*, *P. gingivalis*, and *T. forsythia* and previous studies have demonstrated that co-infection with *F. nucleatum* and *P. gingivalis* or *T. forsythia* induced host immune response and provoked alveolar bone loss (Polak et al., 2009; Settem et al., 2012; Chen et al., 2022). The second cluster was composed of *T. denticola, C. rectus, A. actinomycetemcomitans*, and *T. forsythia* which can be correlated with previous studies that concluded that *P. gingivalis*, *C. rectus*, *F. nucleatum*, *A. actinomycetemcomitans*, and *T. denticola* was associated with biofilm formation at the bottom of human periodontal pockets, the so-called “plaque-free zone.” In addition, pregnant women with more than 30% of bleeding had higher quantity of bacteria from the green, orange and red complexes except *F. nucleatum* that was significantly lower. *P. gingivalis* and *T. denticola* was very significantly higher compared to pregnant women with less than 30% of bleeding sites. These results support the “Keystone–Pathogen Hypothesis,” of which *P. gingivalis* plays a key role, indicating that specific bacteria in limited quantities can influence the host immune system and convert the microbiota from symbiotic to dysbiotic to induce inflammatory disorder (Hajishengallis et al., 2012). Specific periodontal pathogens such as the one of the red complex have virulence factors and thus are considered as potential contributors in adverse pregnancy outcomes (Gómez et al., 2020; Radaic and Kapila, 2021). This could be explained because the increase of bleeding periodontal sites would induce hematological dissemination of periodontal pathogens and their products, and subsequently would later lead to an immune/inflammatory reaction in the feto-placental unit (Horliana et al., 2014).

This study has several limitations. Firstly, our research was to focus on 3 months pregnant women with healthy periodontium thus including adults with healthy gums or largely with localized or generalized gingivitis. In fact, this option was guided on the one hand that initial changes from health to plaque-induced gingivitis may not be clinically detectable, raising effective debates about clinical thresholds for defining pathological versus physiological inflammation and by the high prevalence of gingivitis observed in this population (Gare et al., 2023). Secondly, as in any cross-sectional study, our survey of 3 months pregnant women is penalized by the absence of a control group. The results of this research should be considered as a valid report on the status of periodontal pathogens, but limited to the population of pregnant women concerned. The ideal study design would have been to monitor the participating women from pre-pregnancy to 3 months post-pregnancy. Thirdly, saliva gland diseases which could have implications for the microbiota analysis, were not considered. Fourthly, only 9 pathogens according to the green, orange and red Socransky complexes vs. the 5 major complexes were selected (Socransky et al., 1998).

## 5. Conclusion

This study demonstrated that 3 months pregnant women with healthy periodontium presented interdental bleeding and a dysbiotic microbiota with periodontal pathogens from the orange and red complexes of Socransky. Therefore, these women had a high risk of periodontal disease and, consequently, a risk of an unfavorable pregnancy outcome. Preventive oral prophylaxis measures, particularly individual interdental prophylaxis, must therefore be implemented from the very beginning of pregnancy.

## Data availability statement

The raw data supporting the conclusions of this article will be made available by the authors, without undue reservation.

## Ethics statement

The studies involving humans were approved by ethical committee of Dakar (Senegal). The studies were conducted in accordance with the local legislation and institutional requirements. The participants provided their written informed consent to participate in this study.

## Author contributions

FC: Conceptualization, Formal analysis, Methodology, Project administration, Supervision, Validation, Visualization, Writing – original draft, Writing – review & editing. AK: Investigation, Writing – review & editing. V-EL: Writing – review & editing. DB: Conceptualization, Data curation, Funding acquisition, Investigation, Methodology, Project administration, Supervision, Visualization, Writing – original draft, Writing – review & editing.
